# Development of heterocyclic-based frameworks as potential scaffold of 5-HT_1A_ receptor agonist and future perspectives: A review

**DOI:** 10.1097/MD.0000000000038496

**Published:** 2024-06-14

**Authors:** Weihua Yuan, Yanyan Ma, Hui Zhang

**Affiliations:** a School of Acupuncture-Moxibustion and Tuina, Anhui University of Chinese Medicine, Hefei, China; b Gumei Community Health Service Centre, Shanghai Medical College of Fudan University, Shanghai, China.

**Keywords:** 5-HT_1A_ receptor ligand, antidepressant, depression, heterocyclic

## Abstract

As a subtype of the 5-hydroxytryptamine (5-HT) receptor, 5-HT_1A_ receptors are involved in the pathological process of psychiatric disorders and is an important target for antidepressants. The research groups focus on these area have tried to design novel compounds to alleviate depression by targeting 5-HT_1A_ receptor. The heterocyclic structures is an important scaffold to enhance the antidepressant activity of ligands, including piperazine, piperidine, benzothiazole, and pyrrolidone. The current review highlights the function and significance of nitrogen-based heterocyclics 5-HT_1A_R represented by piperazine, piperidine, benzothiazole, and pyrrolidone in the development of antidepressant.

## 1. Introduction

Depression is a common emotional disorder for which the most common features include reduced confidence, disturbance in sleep or appetite, guilt, a lack of interest, feelings of worthlessness, inattention or even suicidal thoughts.^[1–3]^ Serotonin (5-hydroxytryptamine, 5-HT), commonly referred to as 5-HT, represents a crucial neurotransmitter that modulates central nervous system activity and peripheral functions through interactions with 5-HT receptors.^[4]^ Decreased serotonin levels in the synaptic gap are closely associated with the onset of depression and can lead to severe mood disorders.^[5]^ Among the 7 different subtypes of 5-HT receptors (5-HT_1_–5-HT_7_),^[6–10]^ 5-HT_1A_ receptor has been extensively and intensively studied.^[11]^ In particular, this receptor plays an important role in regulating of serotonergic signaling across various central nervous system (CNS) regions: antagonizing presynaptic self-receptors or agonizing postsynaptic heterologous receptors can produce different antidepressant effects, especially activating postsynaptic 5-HT_1A_ receptors will produce rapid antidepressant effects.^[12–14]^ A key challenge in antidepressant research is the identification of compounds that target particular region(s) of the brain involved in rapid antidepressant action to specifically activate their 5-HT_1A_ receptors, while minimizing possible side effects that can arise through the activation of 5-HT_1A_ receptor subpopulations in other areas of the brain. Current data suggest that biased agonists targeting 5-HT_1A_ receptors hold promise as therapeutic compounds involving the 5-serotonin mechanisms as antidepressant candidates.^[15]^

**Figure 1.**
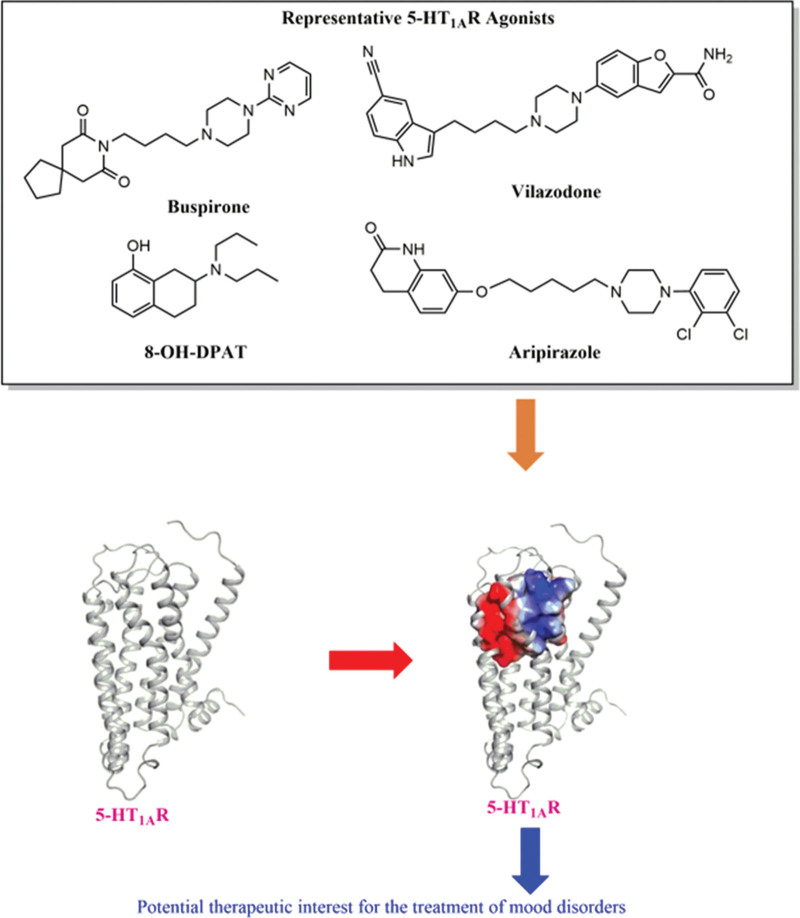
Proposed activation process of 5-HT_1A_R by agonist.

In 5-HT_1A_ receptor ligand design, the heterocyclic scaffold is the most common in the molecule enhances the antidepressant activity of the compound, especially 5-HT_1A_ receptor agonist.^[16,17]^ This review will focus on the medicinal chemistry work that allowed potent and selective 5-HT_1A_R ligands to be identified. In particular, the structure–activity relationships of 5-HT_1A_R ligands represented by piperazine, piperidine, benzothiazole, and pyrroloctone, specifying the pharmacological models and ligand binding modes of 5-HT_1A_R antagonists and agonists (Fig. 1).

## 2. Piperazine

**Figure 2.**
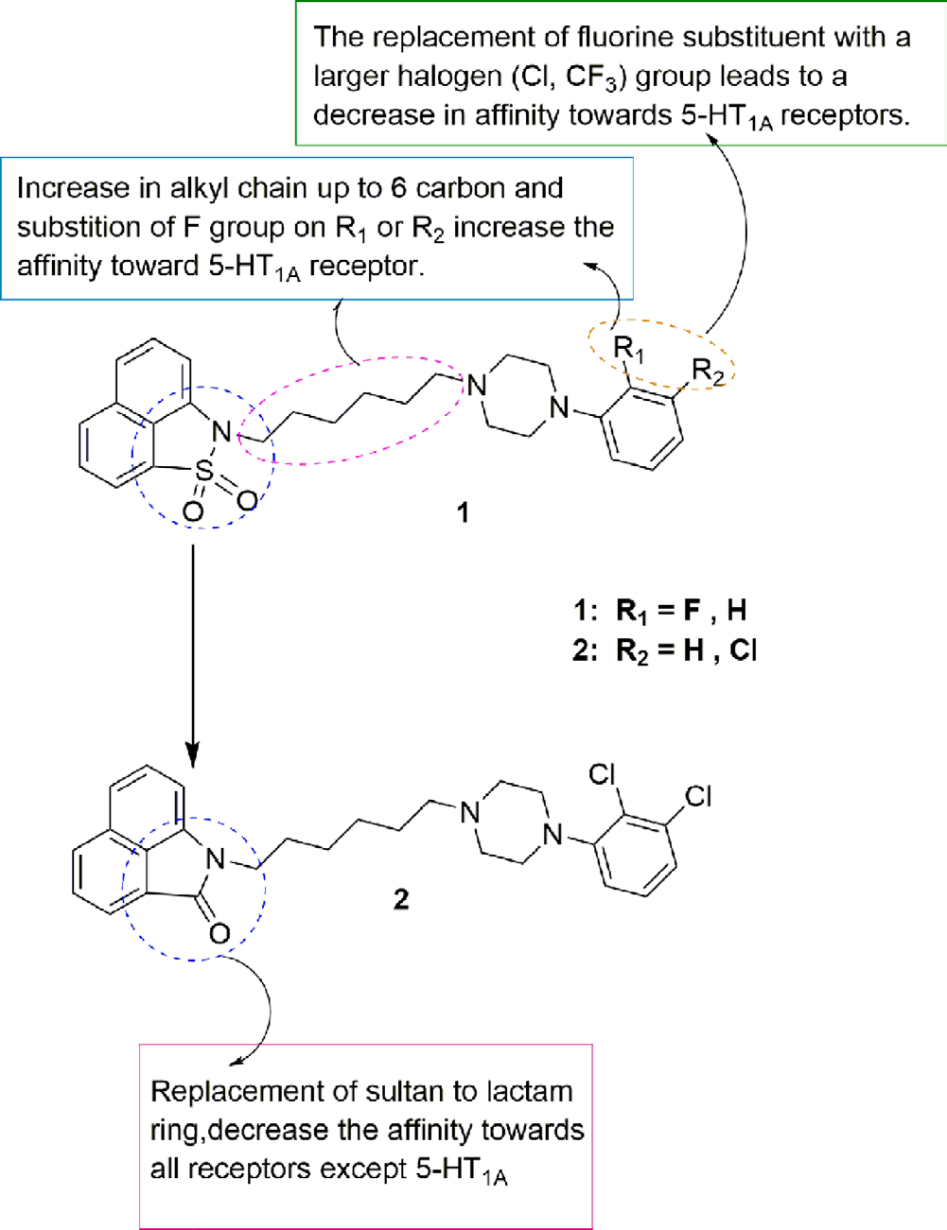
Piperazine-based fananserin derivatives as antidepressants.

**Figure 3.**
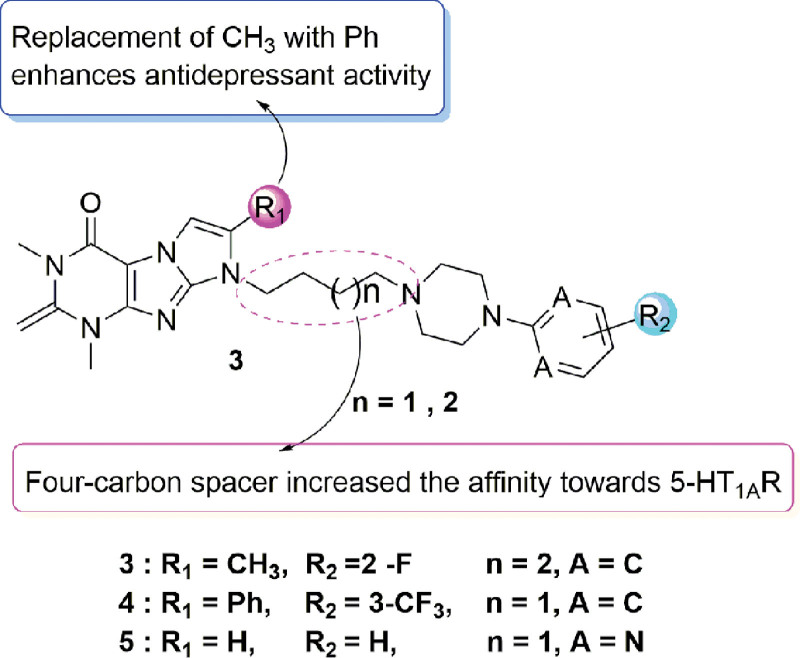
Piperazine-based 1H-imidazo[2,1-f] purine-2,4(3H,8H)-dione derivatives as antidepressants.

**Figure 4.**
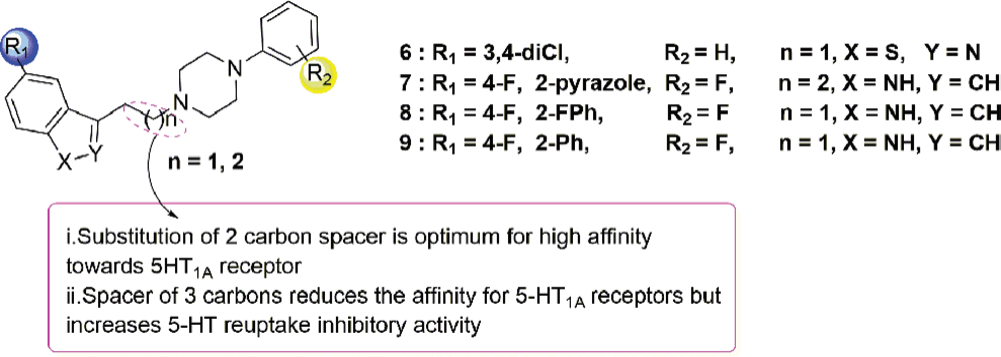
Piperazine-based antidepressants: compounds **6** to **9**.

The piperazine with alkyl chain, a common scaffold in most CNS and neurological disorders compounds with neuroprotective and antidepressant activity,^[18–21]^ is an important group for 5-HT_1A_ agonists. Comprehensive analysis of existing 5-HT_1A_ agonist and antidepression compounds, the piperazine with alkyl chain scaffold present a bridge between 2 heteroaromatic scaffold and where these molecular binding conformations in the 5-HT_1A_. Analysis by computer aided drug design revealed that a CH–π/π interaction with the PHE361 side chain, π–π stacking interactions with PHE362 as well as a salt bridge with ASP116 were important features for stabilizing ligand complexes with the 5-HT_1A_ target.^[22–25]^ Also, piperazines offer a wide range of opportunities to modify and to prove activity and pharmacokinetic profiles. Zareba et al^[26]^ prepared various Fananserin derivatives and evaluated their antidepressant potential. SAR studies showed that increasing the length of the alkyl chain and substituting the 2nd/3rd position of the aryl piperazine with fluorine enhanced ligand affinity to the 5-HT_1A_ receptor. Molecular docking analysis further revealed the formation of hydrogen bonds between the carboxyl group of Asp106 (salt bridge 3.0–3.1 Å) and the nitrogen in the arylpiperazine ring, thus increasing the ligand binding stability to the 5-HT_1A_ receptor (Figs. 2–4).

## 3. Piperidine

**Figure 5.**
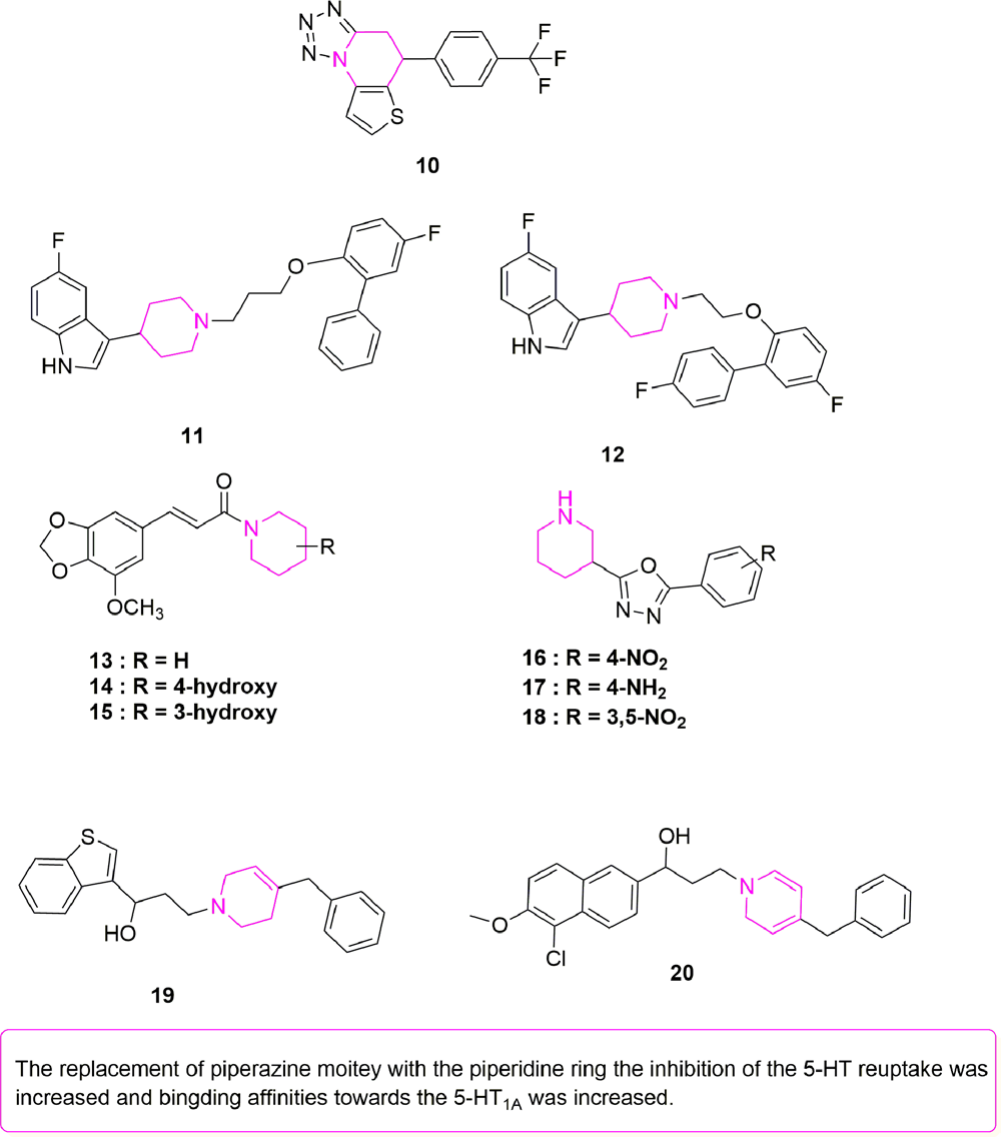
Potent antidepressant activity after replacing piperazine rings with piperidine derivatives: compounds **10** to **20**.

The 6-membered heterocyclic structure of piperidine is commonly found in different naturally occurring bioactive compounds,^[27]^ and studies have revealed its antidepressant potential, along with that of its derivatives.^[28]^ In this context, after synthesizing various 5-aryl-4,5dihydrotetrazolo[1,5-a]thieno[2,3-e]pyridine derivatives that contained tetrazole along with heterocyclic substituents, Wang et al^[29]^ tested their antidepressant-like activity in vivo through TST and FST performed on Kunming mice. In addition to identifying compound **10** (Fig. 5) as having the greatest potency, the results of TST and FST also showed that, at a dose of 40 mg/kg, this compound could achieve a 43.73% and 55.33% reduction in immobility, respectively. Compound **10** or fluoxetine (control) was then administered to the mice before determining the serum level of 5-HT in their brains using enzyme-linked immunosorbent assay (ELISA). In this case, mice from the experimental group had higher amounts of serum 5-HT in their brains compared with those from the fluoxetine group. SAR studies further revealed improved activity and potency when the para-position of the aromatic ring, attached to the piperidine ring’s 4th position, possessed electron-absorbing groups. In a different study, following the synthesis of different alkoxypiperidine derivatives, Wang et al^[30]^ assessed their potential to bind to 5-HT_1A_ receptors as well as to inhibit 5-HT’s reuptake. Of all derivatives, the highest antidepressant activity, assessed through TST, was observed at a dose of 40 mg/kg for compounds **11** and **12** (Fig. 5), with these compounds even showing potent antagonism to 5-HT_1A_ (Ki values: 12 and 17 nM, respectively) as well as moderate 5-HT reuptake inhibition (RUI) (IC_50_ values: 177 and 85 nM, respectively). In vitro studies further suggested that 5-HT_1A_ receptors’ activity was linked to the length of the linkage bond. Therefore, out of various derivatives, compound **10** was identified as the lead compound due to its dose-dependent reduction in the immobility time of mice, as demonstrated through FST and TST, its high potential to inhibit serotonin reuptake (IC_50_ value of 14 nM) and its high affinity for 5-HT_1A_ receptors (Ki value of 12 nM). In addition, animal testing demonstrated good potency and metabolic stability of the compound, while replacing piperazine with piperidine further inhibited serotonin reuptake binding by 5-HT_1A_.^[31]^ Different piperidine and piperazine substituted piperamide congeners were synthesized by Prashanth et al^[32]^ based on the compound’s chemical structure. Although most compounds (at 20 mg/kg) exhibited antidepressant activity during TST and FST, the greatest potency was observed in the case of compounds **13** and **14** (Fig. 5) for which the immobility time decreased by 78.18 and 76.18 seconds, respectively during TST as well as 32.15 and 30.8 seconds, respectively, during FST in comparison with the control. SAR studies have shown that the piperidine moiety is essential for pharmacological activity, with superior antidepressant effects observed when the hydroxyl group occurs at the 4th position of the piperidine ring as opposed to the 3rd position. Different studies have reported the antidepressant effects of 3-[5-(aryl[1,3,4]oxadiazole-2-yl]-piperidine derivatives. In this context, FST results have shown that, in comparison with standard drugs such as tiagabine (10 mg/kg) and imipramine (50 mg/kg) or even a control, 10 mg/kg of compounds **16** to **18** (Fig. 5) could induce a significant reduction in immobility time. This effect was particularly obvious for compound **18**, identified as the most potent, with the immobility time reduced to 103 seconds as opposed to 180 seconds in the case of the control. In contrast, no effects were observed for test compounds in 5-HTP induced head twitches.^[33]^ In order to improve the antidepressant activity of arylalkyl piperazine derivatives, synthesized various arylalkyl piperidine derivatives were prepared by Zheng et al^[34]^ prior to their screening as triple uptake inhibitors. Compared to the positive drug venlafaxine, lower immobility times were noted for compounds **19** and **20** (Fig. 5) in the dose range of 10 to 50 mg/kg during TST. SAR studies further showed greater inhibition of 5-HT, NA, and DA transporters for benzothiophene and 3,4-dichlorobenzene on the aromatic ring attached to the first position of the piperidine ring.

## 4. Benzothiazole

**Figure 6.**
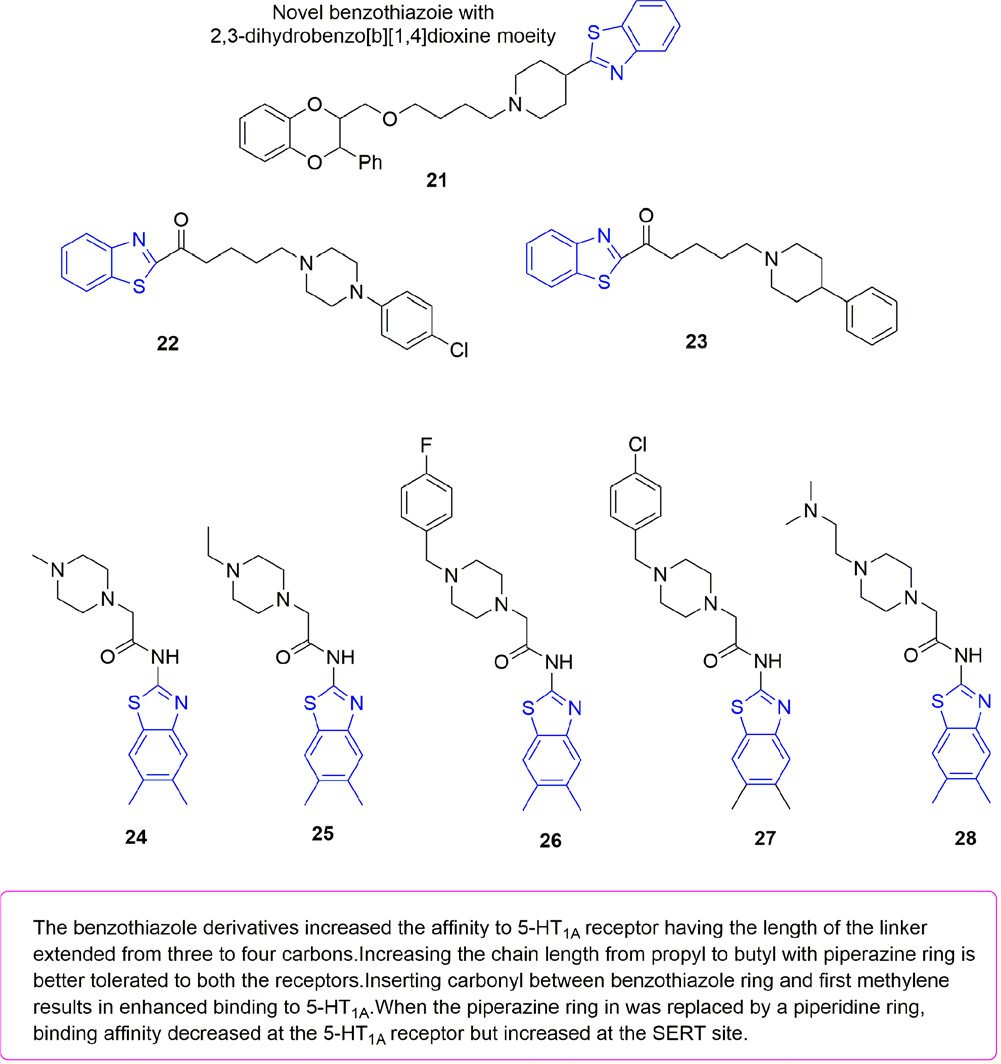
Structure of potent benzothiazole compounds **21** to **28**.

The nucleus of a benzothiazole heterocycle consists of a fused benzene and thiazole motif, with sulfur and nitrogen incorporated within this structure.^[35]^ Benzothiazole derivatives are of significance in CNS-related drugs, such as antidepressants.^[36]^ Compounds containing benzothiazole moieties have the ability to bind to 5-HT_1A_ receptors and SERT transporters and are considered to be the active moiety for antidepressant activity.^[37–39]^ In view of enhancing the efficacy of potential antidepressant compounds, different benzoxazole/benzothiazole derivatives containing 2,3-dihydrobenzo[b}[1,4]dioxine were synthesized by Wang et al^[37]^ before assessing their ability to bind 5-HT_1A_ receptors based on FST and TST assays. Overall, most compounds exhibited binding activity towards 5-HT_1A_ as well as antidepressant effects, with compound **21** (Fig. 6), in particular, displaying the greatest activity. Indeed, with a Ki value of 31 nM, this compound not only displayed optimal binding to 5-HT_1A_ receptors at a dose of 40 mg/kg but also induced a significant reduction in the immobility time of mice. Furthermore, the ED_50_ value of compound **21** in FST compared to the control drug promethazine was found to be 39 mg/kg during a dose–effect relationship study. The results of a SAR study further showed that the activity of compound **21** on 5-HT_1A_ receptors increased as the length of the linkage bond increased from 3 to 4 carbons. Two benzothiazole-based derivatives **22** and **23** (Fig. 6) showed dual binding ability to both SERT and 5HT_1A_ receptor sites. In a different study piperazine was replaced by piperidine in the structure of benzothiazole derivatives, with subsequent SAR analysis indicating reduced binding intensity of 5-HT_1A_ receptors by 2-fold but a 64 nM increase at the SERT site after such structural modifications in compound **23**.^[38]^ To identify fast-acting antidepressant compounds, various benzothiazole derivatives targeting 5-HT_1A_ receptors were also prepared by Demir Özkay et al^[39]^ before orally administering 40 mg/kg of these compounds to mice at 1, 5, and 24 hours prior to TST and MFST. The results indicated that the immobility time of mice decreased significantly for compounds **24** to **28** at the above dose (Fig. 6) compared with the control and standard drug fluoxetine (20 mg/kg).

## 5. Pyrrolidine

Pyrrolidine is a saturated 5-membered heterocycle compound containing a secondary amine.^[40,41]^ Different 3-(1H-indol-3-yl)pyrrolidine-2,5-dione derivatives were synthesized by Wrobel et al^[42]^ prior to their screening to obtain agonist compounds with dual action on SERT and 5-HT_1A_ receptors. SAR studies have revealed the high potency of pyrrolidine-2,5-dione-containing compounds for dual binding to SERT and 5-HT_1A_ receptors, with the piperidine ring having a stronger affinity for the 5-HT_1A_ receptor. In particular, compound **31** exhibited greater 5-HT_1A_ activity in comparison with a serotonin control (Ki value of 3.2 nM vs 2.1 nM). Additionally, in vivo binding assays showed that compounds **29** and **30** could also display binding activity towards receptors other than 5-HT_1A_, thus highlighting their potential as multitargeted antidepressant compounds.

**Figure 7.**
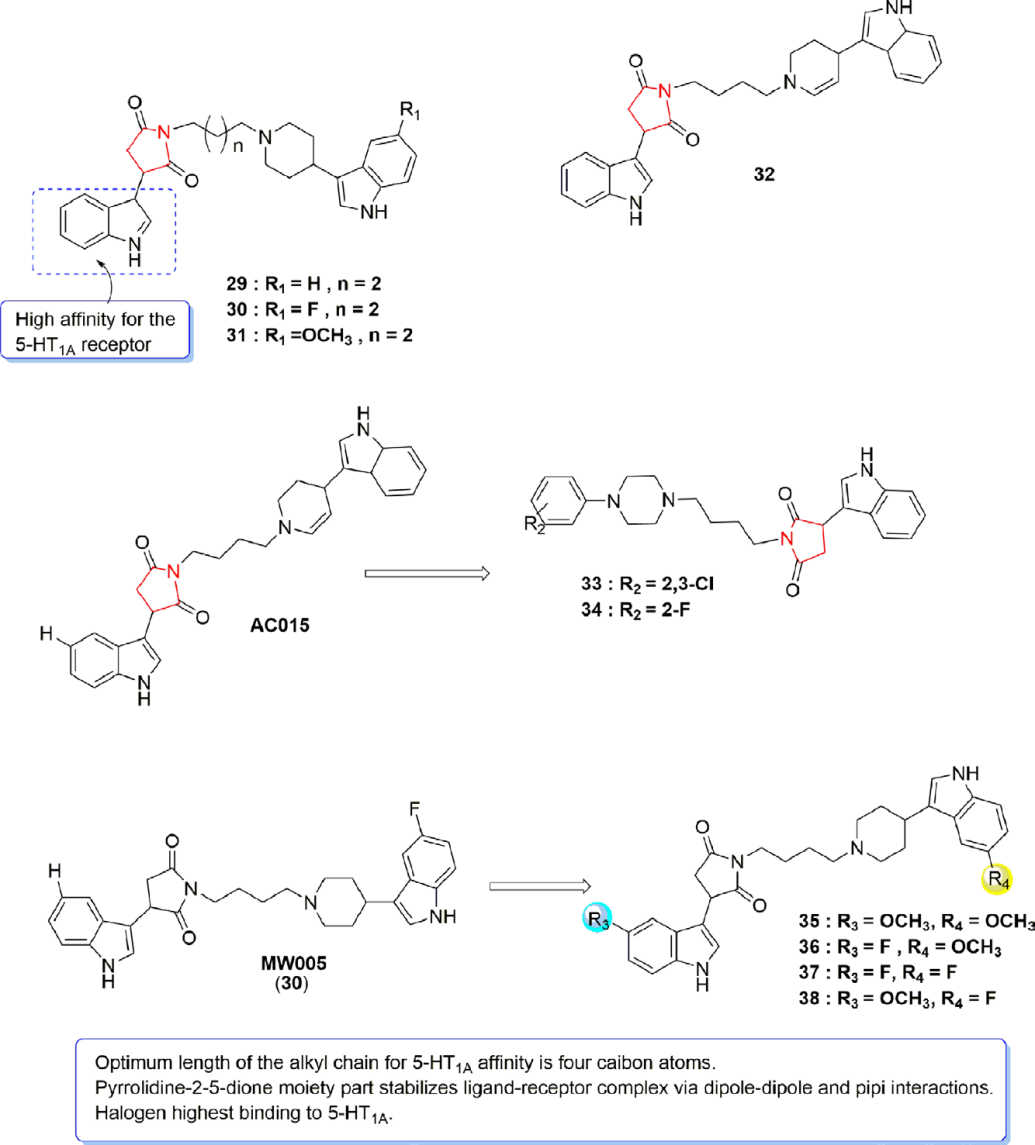
Binding affinity of pyrrolidine-2,5-dione derivatives for 5-HT_1A_ receptor.

Based on this, various 4-butyl-arylpiperazine-3-(1H-indol-3-yl)pyrrolidine-2,5-dione derivatives were designed and prepared by the same group^[43]^ before screening them for 5-HT_1A_/D_2_ receptor binding activity as well as 5-HT reuptake inhibition activity. Among them, compounds **33** and **34** (Fig. 7) exhibited binding activity to 5-HT_1A_ receptors, with higher affinity being especially observed for the former (Ki value: 0.4 nM). However, compound **34** also displayed multitargeting activity, binding to SERT transporters, D_2_ receptors and 5-HT_1A_ (Ki values of 64, 182, and 1.3 nM, respectively). The SAR study further indicated that the o-substituted ligands of phenylpiperazine exhibited higher affinity for 5-HT_1A_R, while the para-substituted compounds showed better affinity for the SERT transporter. Additional evaluation of the binding affinity of 3-(1H-indol-3-yl) pyrrolidine-2,5-dione derivatives for 5-HT_1A_ receptors as well as their inhibitory effects on serotonin reuptake revealed their high affinity for the receptor, with compounds **35** and **36** (Fig. 7) exhibiting the highest activity (Ki values of 2.3 and 3.2 nM, respectively). Compounds **37** and **38** (Fig. 7) also displayed dual binding towards 5-HT_1A_/SERT with Ki values of 7.0 nM (5-HT_1A_) and 17.5 nM (SERT) for compound **37** as well as 4.9 nM (5-HT_1A_) and 17.5 nM (SERT) for compound **38** over 15 days. In vivo FST assays also identified the lead compound MW005 (**30**) as the most active due to its good binding ability to the 5-HT_1A_ receptor and strong antidepressant activity. Therefore compound **30** was identified as the lead compound.

## 6. Conclusion

In this review, we delve into the pivotal role of azacycles in the research and development of antidepressants. Through detailed structure–activity relationship studies, we provide consistent structural characteristic information for the activity of these preclinical molecules. Notably, the piperazine scaffold has emerged as a key platform for antidepressant drug development due to its superior chemical reactivity and CNS pharmacodynamics.

This review highlights the following points: the tunable nature of the piperazine structure, typically consisting of a 3 to 4 carbon chain linking 2 aromatic rings, one of which is attached to the piperazine moiety; the crucial chemical features, including halogen substitution on 1 aromatic ring and hydrogen bond acceptors on the other, which enhance interactions with biological targets. Additionally, we emphasize the significance of other nitrogen-containing heterocycles as ligands for the 5-HT_1A_R. These compounds do not merely bind to 5-HT_1A_R; most of them selectively act on multiple serotonin receptors, thereby exhibiting antidepressant effects.

5-HT_1A_ receptor ligands offer several advantages in antidepressants and neuropsychiatric drugs compared to existing antidepressants. These advantages primarily manifest in the following aspects: multi-target mechanisms: 5-HT_1A_ receptor ligands can bind not only to 5-HT_1A_ receptors but also to various other receptors, such as 5-HT_2A_, 5-HT_2C_, and 5-HT_7_, resulting in multi-target mechanisms. This may contribute to improved efficacy, reduced side effects, and decreased resistance; antianxiety effect: 5-HT_1A_ receptor ligands exhibit strong antianxiety effects, likely due to their agonistic action on 5-HT_1A_ receptors. They may be more suitable for treating patients with depression accompanied by anxiety symptoms compared to existing antidepressants; rapid onset: 5-HT_1A_ receptor ligands demonstrate rapid onset, likely due to their direct agonistic effect on 5-HT_1A_ receptors. They may be more suitable for treating acute depressive episodes compared to existing antidepressants such as selective serotonin reuptake inhibitors (SSRIs); fewer side effects: 5-HT_1A_ receptor ligands tend to have fewer side effects such as sexual dysfunction and weight gain during treatment. This may be attributed to their multi-target mechanisms and regulatory effects on other receptors; less drug-drug interactions: 5-HT_1A_ receptor ligands exhibit fewer interactions with other drugs, potentially improving patient compliance and safety. However, it should be noted that while 5-HT_1A_ receptor ligands offer advantages in antidepressants and neuropsychiatric drugs, more clinical studies are needed to confirm their efficacy and safety. Furthermore, they still face challenges in practical applications, such as drug dosage, administration routes, and individual differences. Therefore, further research and exploration are necessary before applying 5-HT_1A_ receptor ligands in clinical treatment.

Finally, this review focuses on the latest reported compounds with 5-HT_1A_R binding activity, providing guidance for researchers in designing and developing novel nitrogen-containing heterocyclic antidepressants. The aim is to discover drug candidates with stronger binding affinity, higher activity, and lower side effects. Looking ahead, these findings have the potential to not only promote the development of antidepressants but also open new avenues for treating related mental illnesses.

## Acknowledgments

This work was supported by the Natural Science Foundation of Anhui Province (No. 2208085MH272), the Natural Science Research Project of Anhui Educational Committee (Nos. 2023AH050800, KJ2020A0423, and KJ2020A0376).

## Author contributions

**Data curation:** Weihua Yuan, Yanyan Ma.

**Formal analysis:** Weihua Yuan, Yanyan Ma.

**Funding acquisition:** Weihua Yuan, Hui Zhang.

**Methodology:** Weihua Yuan, Hui Zhang.

**Software:** Weihua Yuan, Yanyan Ma.

**Visualization:** Weihua Yuan, Yanyan Ma.

**Writing – original draft:** Weihua Yuan, Yanyan Ma.

**Conceptualization:** Yanyan Ma, Hui Zhang.

**Investigation:** Yanyan Ma, Hui Zhang.

**Project administration:** Hui Zhang.

**Resources:** Hui Zhang.

**Supervision:** Hui Zhang.

**Validation:** Hui Zhang.

**Writing – review & editing:** Hui Zhang.
